# Quantitative Analysis of Burden of Infectious Diarrhea Associated with Floods in Northwest of Anhui Province, China: A Mixed Method Evaluation

**DOI:** 10.1371/journal.pone.0065112

**Published:** 2013-06-06

**Authors:** Guoyong Ding, Ying Zhang, Lu Gao, Wei Ma, Xiujun Li, Jing Liu, Qiyong Liu, Baofa Jiang

**Affiliations:** 1 Department of Epidemiology and Health Statistics, School of Public Health, Shandong University, Jinan City, Shandong Province, P.R. China; 2 School of Public Health, China Studies Centre, The University of Sydney, New South Wales, Australia; 3 National Institute for Communicable Disease Control and Prevention, China CDC, Beijing City, P.R. China; Wadsworth Center, United States of America

## Abstract

**Background:**

Persistent and heavy rainfall in the upper and middle Huaihe River of China brought about severe floods during the end of June and July 2007. However, there has been no assessment on the association between the floods and infectious diarrhea. This study aimed to quantify the impact of the floods in 2007 on the burden of disease due to infectious diarrhea in northwest of Anhui Province.

**Methods:**

A time-stratified case-crossover analysis was firstly conducted to examine the relationship between daily cases of infectious diarrhea and the 2007 floods in Fuyang and Bozhou of Anhui Province. Odds ratios (ORs) of the flood risk were quantified by conditional logistic regression. The years lived with disability (YLDs) of infectious diarrhea attributable to floods were then estimated based on the WHO framework of the calculating potential impact fraction in the Burden of Disease study.

**Results:**

A total of 197 infectious diarrheas were notified during the exposure and control periods in the two study areas. The strongest effect was shown with a 2-day lag in Fuyang and a 5-day lag in Bozhou. Multivariable analysis showed that floods were significantly associated with an increased risk of the number cases of infectious diarrhea (OR = 3.175, 95%CI: 1.126–8.954 in Fuyang; OR = 6.754, 95%CI: 1.954–23.344 in Bozhou). Attributable YLD per 1000 of infectious diarrhea resulting from the floods was 0.0081 in Fuyang and 0.0209 in Bozhou.

**Conclusions:**

Our findings confirm that floods have significantly increased the risks of infectious diarrhea in the study areas. In addition, prolonged moderate flood may cause more burdens of infectious diarrheas than severe flood with a shorter duration. More attention should be paid to particular vulnerable groups, including younger children and elderly, in developing public health preparation and intervention programs. Findings have significant implications for developing strategies to prevent and reduce health impact of floods.

## Introduction

Floods are recognized to be the most frequent and devastating type of natural disaster worldwide [1]. On average, floods and other hydrological events accounted for over 50% of the disasters between 2001 and 2010 in the world [2]. Huaihe River Basin, one of the grain production bases in China, has unique river valley topography. Many tributaries and local weather characteristics in the area bring abundant rainfall and frequent storm floods. The persistent and heavy rainfall in the upper and middle Huaihe River caused several floods during the end of June and July 2007. It was the largest floods since the 1954 Huaihe River floods in this region [3]. In Anhui Province, the floods in 2007 forced an evacuation of thousands of people from homelands, with at least 89 counties and over 15.1 million people affected [4]. The floods hit 1.35 million hectares of crops, of which 0.51 million hectares of crops were demolished. The economic damage was estimated at approximately 10.75 billion Yuan (US$ 1.73 billion) [4].

The health effects of floods are complex and far-reaching, which may include increased mortality and morbidity from diarrheal diseases [5]–[7]. Diarrhea, including infectious (bacteria, parasites, and viruses) and non-infectious (food intolerances or intestinal diseases) diarrhea, remains a major public health problem around the world. Diarrheal disease alone amounts to an estimated 4.1% of the total disability adjusted life years (DALYs) of the Global Burden of Disease (GBD) and is responsible for the deaths of 1.8 million people every year [8]. Water quality may be adversely affected in several ways during floods: contamination of surface or ground water sources by storm water runoff from impermeable or saturated surfaces, introducing fecal contaminants including bacteria, protozoa, and viruses; cross-contamination due to infiltration and inflow between sewage and water pipes, especially in areas with out-of-date water infrastructure; and release of sewage into local waterways because of sewage overflows or bypass [9], [10]. Therefore, there is a potential for increased transmission of infectious diarrhea, a water-borne disease, especially in areas where the population does not have access to clean water and sanitation during floods.

However, the association between floods and diarrheal diseases is far from clear. An increase in diarrheal diseases in post-flood periods has been reported in the 1988 Bangladesh and Khartoum floods [11], [12]. The main risk factors of diarrhea epidemic in the 1998 Bangladesh floods included lack of distribution of water purification tablets and the type of water storage vessels [13]. In high-income countries, the risk of diarrheal illness appears to be lower during floods than that in developing countries. A survey from Germany found that the prevalence of diarrhea was 6.9% in flooded area and the main risk factors were contacting with floodwater, women, and water supply from a private pond [14]. There are also some studies showing no significant increase in risk of diarrhea associated with flooding [15], [16]. With little research has been conducted in China, the effects of the 2007 Huaihe River floods on diarrheal disease remain unknown.

This study aimed to quantify the impact of the floods in 2007 on infectious diarrhea in northwest of Anhui Province, one of the most affected regions in China. Results will contribute to have a better understanding of the health impacts of flooding and assist in developing national strategies to prevent and reduce the burden of infectious diseases associated with floods.

## Materials and Methods

### Ethical Statement

Disease surveillance data used in this study were obtained from the National Notifiable Disease Surveillance System (NDSS) with the approval by Chinese Center for Disease Control and Prevention. All data are unidentified. The present study was approved by the research institutional review board of Public Health of Shandong University.

### Study Areas and the Floods

**Figure 1:**
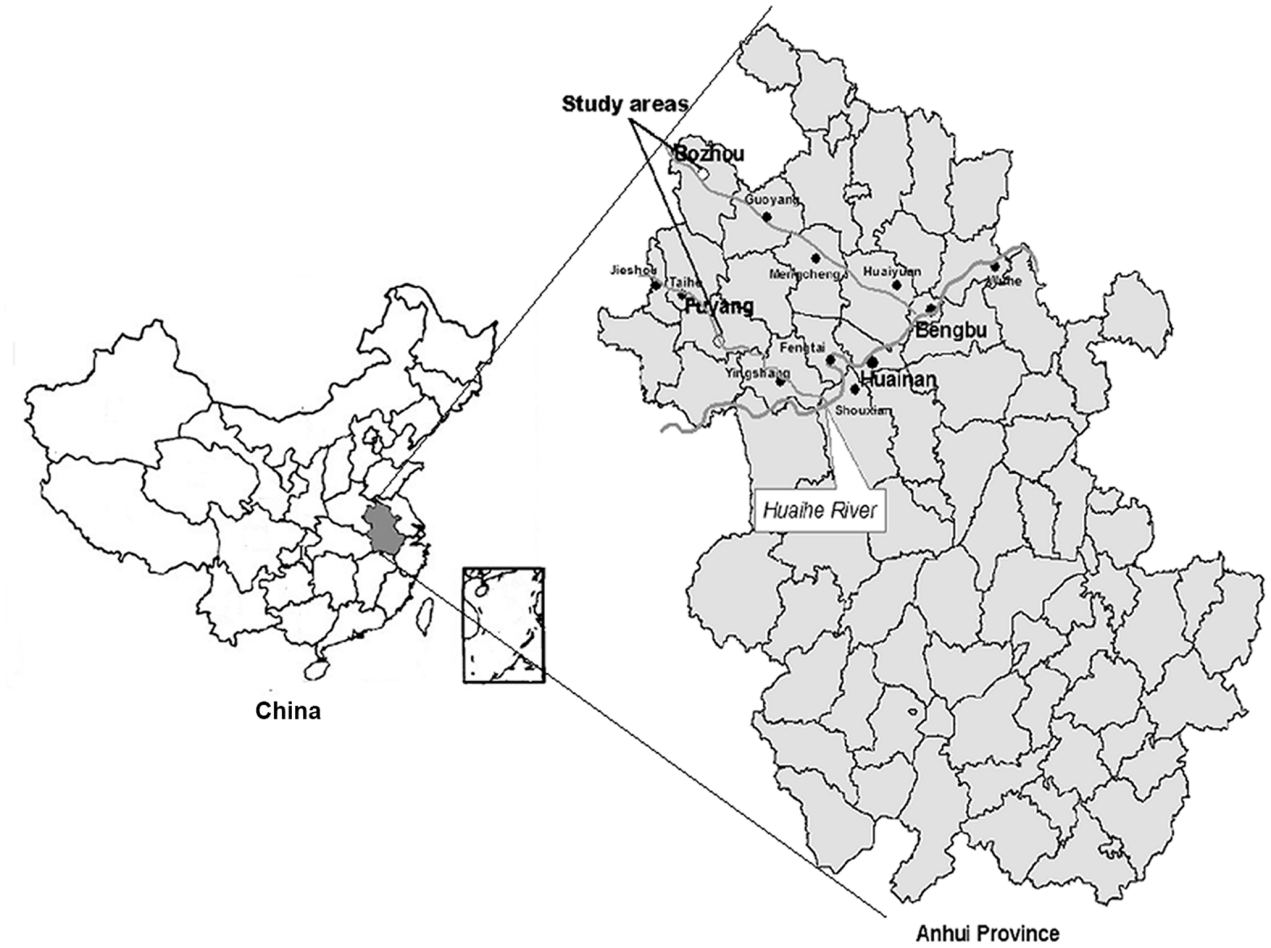
Location of study areas in Anhui Province, China.

**Figure 2:**
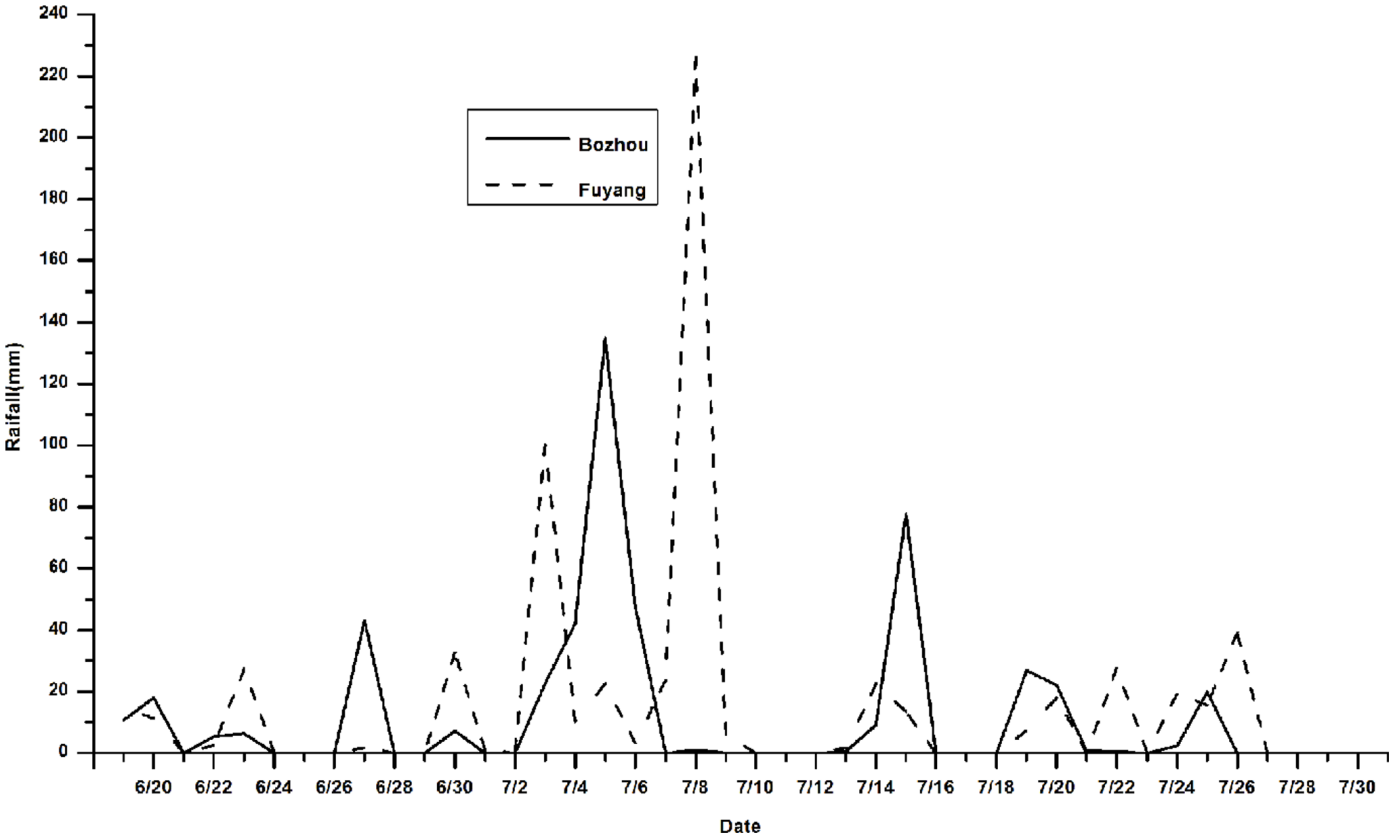
Daily rainfalls in Fuyang and Bozhou during the Meiyu-flood-season, 2007.

During the Meiyu-flood-season of 2007, Huaihe River Basin was hit by severe heavy rainfall with the heaviest precipitation occurring in the north-central Anhui. Our study areas cover Fuyang and Bozhou, two of the worst hit areas, located in the northwest of Anhui Province (Figure 1). Figure 2 shows distribution of daily rainfall in Fuyang and Bozhou during the Meiyu-flood-season of 2007. The total precipitation in Fuyang was 425.9 mm from 30 June to 9 July; and Bozhou had received 297.6 mm from 27 June to 6 July and 87.6 mm from 13 July to 15 July, respectively. According to the flood classification defined by the Comprehensive Study Group of Major Natural Disasters of the State Science and Technology Commission in China, cumulative rainfall of more than 80 mm for three consecutive days or 250 mm for ten consecutive days is a moderate flood; cumulative rainfall of more than 150 mm for three consecutive days or 350 mm for ten consecutive days is a severe flood. According to the flood classification above, Fuyang had suffered one severe flooding and Bozhou had suffered two moderate flooding.

The study areas have sub-humid warm temperate continental monsoon climate. Population in the two study areas was 1,806,339 in Fuyang and 1,410,332 in Bozhou in 2007, accounting for 5.3% of whole population in Anhui Province. The population structure of age, gender and economic development level were similar in the two selected study areas [17].

### Data Collection and Management

#### Disease surveillance data

Daily disease surveillance data on infectious diarrhea from May to September 2007 were obtained from the NDSS. The definition of infectious diarrhea from the NDSS is a group of human diseases that are mainly caused by microbes (including bacteria, parasites, and viruses) and have diarrhea as the typical symptom, including cholera, dysentery, typhoid, paratyphoid and other infectious diarrhea. No new case of cholera, typhoid and paratyphoid was notified during the Meiyu-flood-season of 2007. All infectious diarrhea cases were diagnosed by clinical symptoms as well as the serological test confirmation. The disease surveillance data used in this study were unidentified. Information of cases included age, gender, residential address, type of disease, date of onset, and date of death. According to the National Communicable Disease Control Act, physicians in hospitals must report every case of infectious diarrhea to the local health authority. Then, the local health authority must report these cases to the next level of the organization within 24 hours [18]. Therefore, it is believed that the degree of compliance in disease notification over the study period was consistent. Demographic data were obtained from the Center for Public Health Science Data in China (http://www.phsciencedata.cn/).

#### Meteorological data

Daily meteorological data from May to September in 2007 were collected from the China Meteorological Data Sharing Service System (http://cdc.cma.gov.cn/). The meteorological variables included daily average temperature (AT), daily minimum temperature (MiT), daily maximum temperature (MaT), daily average relative humidity (ARH), daily minimum relative humidity (MiRH), daily average air pressure (AAP), daily minimum air pressure (MiAP), daily maximum air pressure (MaAP), daily average wind velocity (AWV), daily maximum wind velocity (MaWV), daily rainfall (RF), daily average vapor pressure (AVP), and daily sunshine duration (SD).

#### Study design and statistical analysis

A mixed method evaluation was conducted. Firstly, the time-stratified case-crossover design, a study design for assessing the effects of a transient exposure on the subsequent risk, was adopted to quantify the risks of floods. The time-stratified design is not subject to bias resulting from time trend and can control for day of the week by restricting referents to the same day of the week, month, and year as the index day [19]. In addition, the time-stratified design avoids overlap bias associated with a standard conditional logistic regression analysis [20].

The number of new cases of infectious diarrhea was selected as an index of the acute effect caused by floods. The exposure periods were flooding periods in these two areas, i.e. one flood (30 June to 9 July) in Fuyang, and 2 floods (27 June to 6 July, and 13 July to 15 July) in Bozhou. We used the time-stratified approach to select control days [21]. Thus, every seventh day from the event day within the same month and year of the event day was considered a control day. If one exposure day was chosen as a control day, this day was excluded as control days. Therefore, one exposure day was matched with three or four control days. Fuyang had 10 exposure days and 32 control days, and Bozhou had 13 exposure days and 41 control days.

After a descriptive analysis, the Wilcoxon Two-Sample test was applied to examine the difference in the number of daily cases of infectious diarrhea and meteorological variables between the exposure and control periods. With the consideration of potential lagged effects of the floods, lagged effects (up to seven days) were assessed by conditional logistic regression analysis. Odds ratios (ORs) and 95% confidence intervals (CI) of the floods on the number of incident cases of infectious diarrhea were calculated in each model. After fitting the lags, we adjusted for temperature, humidity pressure, wind velocity, vapor pressure and sunshine duration in the multivariate conditional logistic regression models. ORs and 95%CI of infectious diarrhea due to exposure of the floods were estimated. All statistical analyses were performed using SAS 9.1.3 (SAS Institute Inc., USA).

Secondly, the potential impact fraction (PIF) was estimated based on the environmental framework of comparative risk assessment (CRA) developed by the WHO. The burden of disease attributable to a specified change in level of a risk factor was estimated using the formula for the PIF [22]. This formula applies to any situation in which a population is distributed into graded levels of exposure.
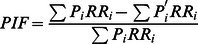
Where: PIF = Potential impact fraction. P_i_ = Proportion of the population in exposure category i. 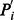 = Proportion of the population in exposure category i after an intervention or other change. RR_i_ = Relative risk at exposure category i compared to the reference level.

The counterfactual level could be the minimum disease burden achievable in a given time frame. If exposed populations were to be compared with unexposed populations, the burden of disease reduction can be calculated from a simplified form of the above formula [23]:
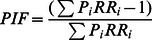



The relative risk (RR) can be best estimated using a population sample, but if the rare disease assumption holds, OR is a good approximation to RR [24]. So:
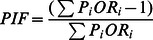



Based on the estimates of ORs above, we calculated PIF of infectious diarrhea due to the floods.

Thirdly, years lived with disability (YLDs) and attributable YLDs were calculated to estimate the disease burden of infectious diarrhea due to the floods. Since there was no death of infectious diarrhea notified during the study period, we adopted the YLDs to assess disease burden of infectious diarrhea. We collected cases of infectious diarrhea during the hazard period of the floods. The method to estimate the YLDs as recommended by the WHO was used to assess the disease burden of infectious diarrhea [25]. Calculations of YLDs and YLD per 1000 were made using DISMOD II (WHO, 2001; a computer software program developed for GBD that allows the user to check if a set of assumptions on incidence, prevalence, remission, case-fatality rates and observed mortality numbers are consistent with one another [25]) and Microsoft Office Excel 2003 (Microsoft Corp., USA). To calculate the fraction of infectious diarrhea attributable to the floods for the study population, the YLDs for the population was multiplied by PIF. The equation is [23]:
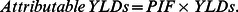



## Results

### Descriptive Analysis for the Disease and Meteorological Data (Tables 1, 2 and 3)

A total of 197 infectious diarrheas were notified in the two study areas over the study exposure and control periods. Majority of the patients were male (63.96%) with 59.90% aged below 14 years, 32.49% between 15 and 59 years, and 7.61% over 60 years. Tables 1 and 2 display a summary of the distribution of daily data of diarrhea cases and meteorological variables in the study areas. The distribution of the number of daily cases of infectious diarrhea, RF and ARH were significantly different between the exposure and control periods in Fuyang (p<0.05). The daily number of cases of infectious diarrhea, RF, AAP, MiAP, and MaAP in the exposure period were significantly different to those in the control period in Bozhou (p<0.05). Other meteorological variables differed slightly across the two periods in the study areas.

**Table 1:** Distribution of daily data on cases of infectious diarrhea and meteorological variables in Fuyang.

	Period	Mean±*SD*	Min	P_25_	Median	P_75_	Max
Cases ofdiarrhea	Control	1.62±1.17	0	0.50	1	2.25	5
	Exposure*	3.20±1.93	1	2	3	4	8
AT (°C)	Control	26.46±2.83	21	24	27	29	31
	Exposure	25.00±1.56	23	24	24.50	26.25	28
MiT (°C)	Control	23.19±2.50	18	21.75	23	25.25	28
	Exposure	22.90±1.20	21	22	23	24	25
MaT (°C)	Control	30.54±3.72	24	27	31	34	36
	Exposure	28.50±2.55	26	26	28	31.25	32
ARH (%)	Control	85.73±8.10	65	82.75	86	93	97
	Exposure*	92.50±5.74	82	88.50	93.50	97.25	99
MiRH (%)	Control	66.81±13.41	36	60.75	66.50	75.75	90
	Exposure	77.50±14.73	51	66.25	83.50	87.75	93
AAP (hPa)	Control	999.73±3.68	993	996.50	1001	1003	1004
	Exposure	997.40±1.65	994	996	998	998	1000
MiAP (hPa)	Control	997.88±3.79	991	994	999	1001	1002
	Exposure	995.80±1.75	992	994.75	996	997	998
MaAP (hPa)	Control	1001.23±3.66	995	997.75	1002.5	1004.25	1005
	Exposure	999.00±1.63	995	998	1000	1000	1000
AWV (m/s)	Control	2.46±0.90	1	2	2	3	4
	Exposure	1.80±0.63	1	1	2	2	3
MaWV (m/s)	Control	4.85±1.59	2	4	5	6	8
	Exposure	5.20±1.55	9	4	5	6.25	8
RF(mm)	Control	7.42±11.57	0	0	0	16.50	39
	Exposure*	42.60±70.92	0	2.50	16.50	49.75	226
AVP (hPa)	Control	29.50±4.71	19	26.75	30	34	35
	Exposure	29.30±1.34	27	28.75	29	31	31
SD (h)	Control	4.58±4.66	0	0	4	10	11
	Exposure	1.50±2.17	0	0	0	3.25	6

*SD*: standard deviation; Min, minimum; P_25_, the 25th percentile; P_75_, the 75th percentile; Max, maximum.

AT, average temperature; MiT, minimum temperature; MaT, maximum temperature; ARH, average relative humidity; MiRH, minimum relative humidity; AAP, average air pressure; MiAP, minimum air pressure; MaAP, maximum air pressure; AWV, average wind velocity; MaWV, maximum wind velocity; RF, rainfall; AVP, average vapor pressure; SD, sunshine duration.

* p<0.05 vs. Control.

**Table 2:** Distribution of daily data on cases of infectious diarrhea and meteorological variables in Bozhou.

	Period	Mean±*SD*	Min	P_25_	Median	P_75_	Max
Cases ofdiarrhea	Control	2.11±1.83	0	0.25	2	3	6
	Exposure*	3.62±2.33	0	2	3	5	9
AT (°C)	Control	26.33±3.55	20	24	26	29.75	32
	Exposure	25.77±2.45	22	24	26	27.50	31
MiT (°C)	Control	22.81±2.77	18	21	22.5	25	28
	Exposure	22.69±2.02	20	20.50	23	24	26
MaT (°C)	Control	30.72±4.53	23	26.25	32	35	38
	Exposure	29.46±3.60	23	27	30	32	36
ARH (%)	Control	72.25±13.50	45	63	75	83.75	89
	Exposure	78.38±9.11	64	68	80	86	90
MiRH (%)	Control	54.22±18.83	15	41.5	56	69	82
	Exposure	61.00±16.06	64	68	80	86	90
AAP (hPa)	Control	999.97±3.47	992	998	1002	1002.75	1004
	Exposure*	997.38±1.04	995	997	998	998	999
MiAP (hPa)	Control	998.11±3.87	994	995.25	1000	1001	1002
	Exposure*	995.31±1.78	992	994.5	996	996.5	998
MaAP (hPa)	Control	1001.58±3.32	994	999	1003	1004	1006
	Exposure*	998.85±0.90	997	998	999	999.5	1000
AWV (m/s)	Control	2.19±0.79	1	2	2	3	4
	Exposure	2.00±0.58	1	2	2	2	3
MaWV (m/s)	Control	4.14±1.25	2	3	4	5	8
	Exposure	4.85±1.41	2	4	5	6	7
RF(mm)	Control	2.83±7.00	0	0	0	0.75	27
	Exposure*	29.69±40.23	0	0	9	45.50	135
AVP (hPa)	Control	24.36±5.02	16	19	25.5	28	33
	Exposure	25.23±2.09	23	23.5	25	27.5	29
SD (h)	Control	4.44±4.17	0	0	4	8.75	11
	Exposure	3.38±4.50	0	0	1	8	11

*SD*: standard deviation; Min, minimum; P_25_, the 25th percentile; P_75_, the 75th percentile; Max, maximum.

AT, average temperature; MiT, minimum temperature; MaT, maximum temperature; ARH, average relative humidity; MiRH, minimum relative humidity; AAP, average air pressure; MiAP, minimum air pressure; MaAP, maximum air pressure; AWV, average wind velocity; MaWV, maximum wind velocity; RF, rainfall; AVP, average vapor pressure; SD, sunshine duration.

* p<0.05 vs. Control.

During the flood-period, 102 cases of diarrhea were identified by the local relevant laboratories. Of these, 66 (64.71%) and 36 (35.29%) had a dysentery and other infectious diarrhea, respectively. As shown in Table 3, the incidence rates of infectious diarrhea in Fuyang and Bozhou were 2.048/10^5^ and 4.609/10^5^, respectively. The incidence rates of male in the study areas were significantly higher than those of female (2.275/10^5^ vs. 1.812/10^5^ in Fuyang, and 5.912/10^5^ vs. 3.221/10^5^ in Bozhou). The incidence rates of infectious diarrhea were highest in children under 4 years of age (5.257/10^5^ in Fuyang, and 38.541/10^5^ in Bozhou).

**Table 3:** Incidence rate of infectious diarrhea among age-sex groups in the flood-period (1/10^5^).

	Fuyang			Bozhou		
Age group (years)	Male	Female	Total	Male	Female	Total
0–4	7.212	3.134	5.257	50.008	26.081	38.541
5–14	0.764	1.852	1.256	8.703	5.064	7.127
15–29	1.927	0.815	1.386	1.547	1.114	1.338
30–44	2.408	1.628	2.021	0.943	0.996	0.969
45–59	0.819	3.324	2.062	1.071	1.083	1.077
60–69	1.867	1.960	1.912	0.000	0.000	0.000
70–79	6.389	2.782	4.461	4.239	0.000	1.885
80+	0.000	0.000	0.000	34.513	9.638	18.552
Total	2.275	1.812	2.048	5.912	3.221	4.609

### Analysis for Lagged Effects (Figure 3)

**Figure 3:**
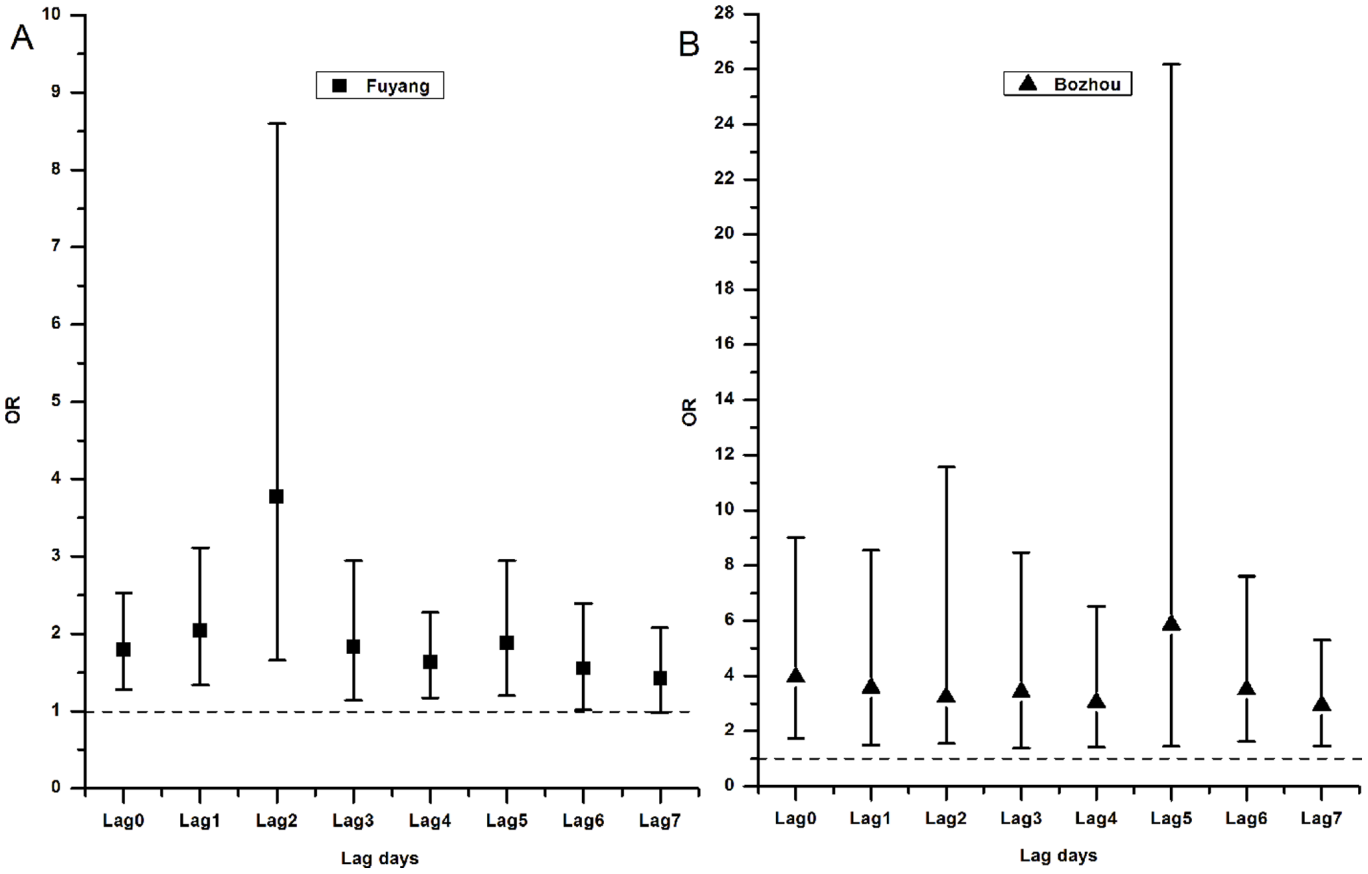
OR estimates of the floods on the risk of infectious diarrhea in different lagged days.

Figure 3 shows the estimated ORs of the floods on the risk of infectious diarrhea on various lagged days. Floods significantly increased the number of cases in Fuyang (ORs>1 from lag 0 to lag 6) and Bozhou (all ORs>1 on different lagged days). The strongest effect was observed at lag 2 days in Fuyang (OR = 3.774, 95%CI: 1.659–8.598), and lag 5 days in Bozhou (OR = 5.859, 95%CI: 1.448–26.170), respectively. These lagged effects were incorporated in the next multifactorial regression analysis.

### Conditional Logistic Regression Analysis (Table 4)

Parameters estimates and test results of multifactorial conditional logistic models in the two areas are presented in Table 4. Multivariable analysis showed that the floods were significantly associated with an increased risk of infectious diarrhea after adjusting for other meteorological factors (OR = 3.175, 95%CI: 1.126–8.954 in Fuyang; OR = 6.754, 95%CI: 1.954–23.344 in Bozhou).

**Table 4:** Parameters of the variables which were significantly included in the multifactorial conditional logistic models in the two areas.

Area	Variable	Estimate	*SE*	Wald χ^2^	p-value	OR	95%CI
Fuyang	Flood	1.155	0.529	4.768	0.029	3.175	1.126–8.954
	AWV	−1.255	0.608	4.264	0.039	0.285	0.087–0.938
	AAP	−0.507	0.229	4.886	0.027	0.603	0.385–0.944
Bozhou	Flood	1.910	0.633	9.113	0.003	6.754	1.954–23.344
	MaWV	1.415	0.668	4.482	0.034	4.116	1.111–15.256
	MiAP	−0.260	0.113	5.313	0.021	0.771	0.618–0.962

*SE*, standard error; OR, odds ratio; CI, confidence intervals.

AWV, average wind velocity; AAP, average air pressure; MaWV, maximum wind velocity; MiAP, minimum air pressure.

Proportion of the population exposed to the floods in Fuyang and Bozhou was 0.438 and 0.562, respectively. Based on the above formula for PIF, we calculated that PIF of infectious diarrhea due to the floods was 0.807.

### Analysis for YLDs and Attributable YLDs of Infectious Diarrhea (Table 5, Figure 4)

YLD per 1000 of infectious diarrhea among age-sex groups in the flood-period is presented in Table 5. The total YLD per 1000 in Bozhou was significantly higher than that in Fuyang (0.0259 vs. 0.0100). The YLD per 1000 of female in Fuyang was higher than that of male (0.0065 vs. 0.0035), while the opposite was the case in Bozhou (0.0061 vs. 0.0198). The YLD per 1000 of infectious diarrhea in Fuyang was highest between the ages of 70 and 79 years (0.0275), following by the age of 60–69 (0.0145). While the highest YLD per 1000 in Bozhou was in children less than 4 years of age (0.2441), the second was in people aged 80 years or older (0.0609).

**Table 5:** YLD per 1000 of infectious diarrhea among age-sex groups in the flood-period.

	Fuyang			Bozhou		
Age group(years)	Male	Female	Total	Male	Female	Total
0–4	0.0086	0.0037	0.0122	0.2139	0.0302	0.2441
5–14	0.0013	0.0056	0.0069	0.0170	0.0076	0.0246
15–29	0.0031	0.0014	0.0045	0.0027	0.0029	0.0055
30–44	0.0046	0.0097	0.0143	0.0034	0.0040	0.0074
45–59	0.0013	0.0071	0.0083	0.0033	0.0088	0.0121
60–69	0.0024	0.0122	0.0145	0.0000	0.0000	0.0000
70–79	0.0081	0.0193	0.0275	0.0029	0.0000	0.0029
80+	0.0000	0.0000	0.0000	0.0549	0.0060	0.0609
Total	0.0035	0.0065	0.0100	0.0198	0.0061	0.0259

**Figure 4:**
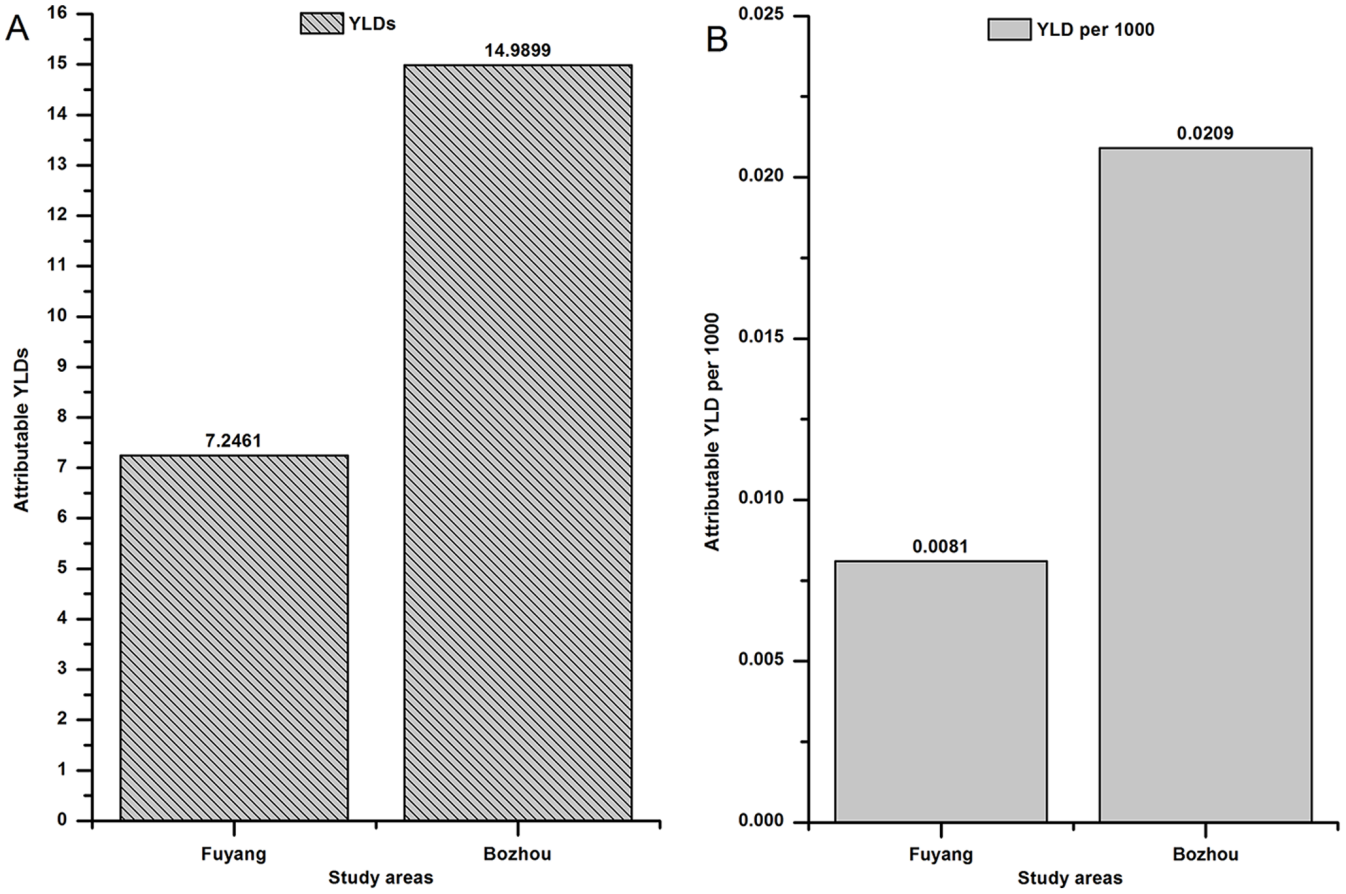
Attributable YLDs and YLD per 1000 among the two areas in the flood-period.

Figure 4 shows the attributable YLDs and YLD per 1000 in the two areas over the flood-period. The attributable YLDs and YLD per 1000 in Bozhou (14.9899, 0.0209) were significantly higher than those in Fuyang (7.2461, 0.0081).

## Discussion

This study has, for the first time, quantified the association between floods and infectious diarrhea in northwest of Anhui Province, China. The study confirms that flood hazard will bring more cases of infectious diarrhea than non-hazard periods in the study areas. Although the case study is based on only two areas in Anhui Province (the most affected), the real burden of infectious diarrheal due to flood will be much higher than the estimates from this study, given the larger population at risk of diarrheal disease in China.

An increased risk of diarrheal disease following floods has been reported in both developing countries [26]–[28] and developed countries. For example in developed countries, during the 2001 floods in Texas, flooded households were associated with a greater risk of diarrhea than non-flooded homes (OR = 10.8, p<0.01) [29]. Another study from the United States revealed that an increase in the incidence of diarrhea during the flood was observed (incidence RR = 1.29, 95%CI: 1.06–1.58), and this effect was pronounced among persons with potential vulnerability to infectious diarrhea [30]. A German study also showed that the major risk factor for diarrhea was contact with floodwater (OR = 5.8, 95%CI: 1.3–25.1) [14]. In our study, the analysis shows that flooding has contributed to an increased risk of infectious diarrheas in the flooded areas in China. Analysis from this study shows that nearly two times increase in average daily number of diarrheal cases was observed in both Fuyang and Bozhou during the 2007 floods. Heavy rainfall may cause flooding and changes in living environment. Floodwaters are able to foster the growth of many pathogens and lead to a lack of clean water and food supply. In addition, damage of the sewage system during floods can increase the transmission of communicable diseases [6], [31]. The reason for a high percentage of dysentery, including bacillary dysentery and Amebic dysentery, among all the diarrheal cases cannot be identified from our study. It may be due to the local environment conditions that are suitable for the pathogen and the spread of the disease or a lower rate of under-reporting in Anhui Province, where the two study areas located [32].

This study has identified a larger effect of floods on infectious diarrhea in Bozhou with a longer lagged effect than in Fuyang, which suggests that burden of disease due to infectious diarrhea caused by riverine floods (with heavy and long-duration rainfall) is relatively more severe than that by flash floods (with relatively short intense bursts of rainfall). During the flood period, rainfall may affect the frequency and level of contamination of drinking water [33]. The total precipitation of Fuyang was higher than that of Bozhou during flood hazard days. But Fuyang had the characteristics of concentrated rainfall within a shorter period of time, even one-day maximum precipitation amount of 226.1 mm. In Bozhou, the flood hazard periods last longer. The water receded very slowly, changing the earth into a flowing river of mud and creating vast of stagnant water in Bozhou. Therefore, a long hazard period can lead to excessive flooding break down water and exacerbate sanitation systems and promote the intake of contaminated drinking water [34], [35]. The other factors that may affect the lagged effect include the growth of pathogens under suitable environmental conditions that cause diarrhea, spread through contaminated food or water and other health infrastructures [36]. It also creates more opportunities for individuals in Bozhou than those in Fuyang to contact with floodwater. Our findings support that prolonged moderate flooding may cause more burdens of infectious diarrheas than severe flooding with shorter duration.

To estimate burden of disease, we adopted a comprehensive measurement-YLDs, which can assist in evidence-based allocation of limited health resources. In an urban case study in the UK, DALYs were used to quantify of health effects of flooding, and the YLDs of gastrointestinal illness were 0.0010 and 0.0006 in their two study areas, which is the first attempt to quantify a wide range of health impacts resulting from flooding using DALYs [37]. However, the burden of gastrointestinal illness that can be attributed to the flood event was not estimated in that study. The incidence rate of male in Fuyang was higher than that of female, while the YLD per 1000 of female in Fuyang was higher than that of male, which suggests that female may suffer more burden of disease than male in Fuyang. It is not clear whether the difference in disease burden between male and female is caused by different response behaviors. A possible explanation is that in the moderate flooding, males participated in more relief work and engaged more frequently than females, leading to a higher exposure for males in Bozhou [38]. While in the severe flooding, all people participated in relief work and females and males had similar exposures to adverse environment. In this scenario, flooding may bring more health risks to women than men. In terms of the age-groups, it suggests that younger children and elderly are the most vulnerable groups to have infectious diarrhea associated with floods. It is better to use YLDs than incidences, which only consider the number of survived persons but ignoring the quality of life, to show the impact of floods.

There are some advantages in our mixed approach. Firstly, we have adopted the time-stratified case crossover design that allows unbiased conditional logistic regression estimates, and avoids bias resulting from time trend in the exposure series, and can be tailored to match on specific time-varying confounders [19]. Secondly, we have controlled other meteorological factors in the regression models with consideration of lagged effects of the floods. Thirdly, YLDs caused by floods in China have been estimated for the first time by this study. Methods could be borrowed and validated by other studies in this field.

There are some limitations in our study. One of the main limitations is that there are many factors can affect the transmission of infectious diarrhea, e.g. human activities, socio-economic status, availability of health services, environmental hygiene, which could not be included in this analysis. However, it only has minimal effect on the comparison between the two study areas as these conditions are similar in these areas. In addition, only two study areas in Anhui are selected in the analysis. More studies in other flood affected regions in China with different socio-economic and weather conditions should be conducted to have a better understanding of the health impact of floods.

### Conclusion

This study has, for the first time, quantified the impact of floods on infectious diarrhea in China using a mixed method approach. Results suggest that floods can significantly increase the risks of infectious diarrhea in the study areas with various lagged effects. In addition, prolonged floods may bring more burdens of infectious diarrhea than flash floods with a shorter period. More attention should be paid to particular vulnerable groups, including younger children and elderly, in developing public health preparation and intervention programs. Findings have significant implications for developing strategies to prevent and reduce health impact of floods.
